# Bail Out of Complications during Endovascular Surgery

**DOI:** 10.3400/avd.ra.26-00062

**Published:** 2026-05-20

**Authors:** Koji Maeda

**Affiliations:** Department of Vascular Surgery, International University of Health and Welfare, Narita Hospital, Narita, Chiba, Japan

**Keywords:** endovascular therapy (EVT), bail out, complications, bleeding, dissection, rupture

## Abstract

Serious complications due to remote control often may occur during endovascular surgery. Therefore, it is necessary to know what complications are likely to occur or how to bail out those complications. Herein, we explain complications related to endovascular surgery and their bail-out procedures. (This is a translation of Jpn J Vasc Surg 2024; 33: 131–136.)

## Introduction

Endovascular therapy (EVT) is flourishing as a treatment for aortic and peripheral arterial diseases. In recent years, as indications for venous stents have also expanded, demand for EVT is predicted to increase further. However, EVT is a remote operation, and unexpected problems may occur. Because it is a remote operation, it can sometimes lead to serious complications, and responses different from those in open surgery may be required. Here, we explain the representative complications in EVT and their troubleshooting.

## I. Bleeding

Bleeding is the most frequently encountered complication in EVT, and prompt response is required. Bleeding can be divided into puncture site bleeding and bleeding away from the operator’s hands, such as intra-abdominal or intrathoracic bleeding.

Puncture site bleeding mainly occurs intraoperatively or postoperatively; manual compression is performed first in the former. The concomitant use of duplex ultrasound (DUS) is useful because it enables compression while confirming the bleeding point. However, in high puncture cases that cross the inguinal ligament during a common femoral artery (CFA) approach, hemostasis by manual compression may be difficult, and in such cases, other methods, such as direct hemostasis, should be considered. Although safe hemostasis can be obtained using a hemostatic device, device failure can also occur; therefore, each device should be used after mastering its use.^1)^ Hemostatic devices, such as ProStyle (Abbott Vascular, Santa Clara, CA, USA), Exoseal (Cordis, Miami Lakes, FL, USA), and Angio-Seal (Jude Medical, Minnetonka, MN, USA) have become available.

When a high or low puncture is performed with a CFA approach, or when hemostasis by manual compression is otherwise difficult, a balloon can be placed across the puncture site to puncture the contralateral CFA. Depending on the case, the ipsilateral superficial femoral artery can be punctured and a balloon can be similarly inserted into the bleeding site to achieve hemostasis. After confirming balloon hemostasis by contrast imaging, a small amount of heparin is administered intravenously, the area just above the bleeding site is surgically incised, the puncture site is exposed, and it is directly sutured. An oblique incision is acceptable, but a longitudinal incision can be made if exposure is expected, and the artery can be secured proximal and distal to the bleeding site. During suturing, the balloon is pulled down and deflated, and care is taken to ensure that the balloon does not catch the needle and suture. After suturing, confirmation is performed again by contrast imaging.

**Fig. 1:**
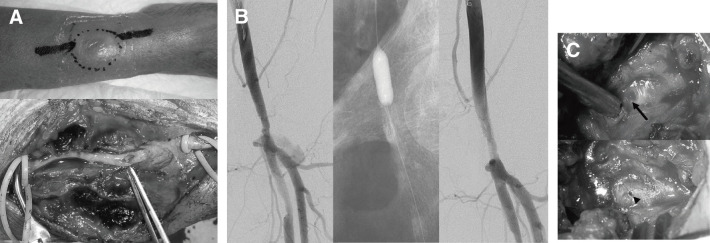
A horizontal incision was performed for the treatment of radial artery pseudoaneurysm (**A**). Hemostasis using a balloon was performed for the common femoral artery pseudoaneurysm (**B**), and suture repair was performed for the pinhole that was created by the insertion of the sheath (**C**).

Even if hemostasis could be achieved intraoperatively, bleeding may occur late due to hemostatic failure or postoperative use of antiplatelet agents or anticoagulants, and a pseudoaneurysm may form in such cases. For the treatment of a pseudoaneurysm, the standard is a method in which hemostasis is achieved with a balloon, the aneurysm is opened, and the bleeding point is surgically controlled (**Fig. 1**). However, if time has elapsed, bleeding may be controlled by securing normal vessels proximal and distal to the bleeding point (at the aneurysm). In this case, a longitudinal incision may provide a better operative field. Furthermore, in the treatment of pseudoaneurysm, hemostasis may be possible by ultrasound-guided thrombin injection; however, the thrombin injection method is an off-label use in Japan, and it must be used cautiously because ethical considerations must also be taken into account.^2)^

To avoid puncture site bleeding, the position of the access vessel must be confirmed by computed tomography (CT) in advance. In particular, in femoral artery approaches, by confirming the position of the femoral head and the bifurcation between the superficial and deep femoral arteries in advance, intraoperative trouble is markedly reduced. In high puncture, bleeding may extend into the retroperitoneum, and intraoperative bleeding may be overlooked; thus, caution is required. In addition, performing a puncture under DUS guidance and confirming hemostasis by DUS after postpuncture compression helps avoid complications.

However, bleeding in the thoracic or abdominal cavity often occurs when large-bore sheaths, such as those for stent grafts, are used. Access injury is the most common, and in this case, massive bleeding often occurs, and shock is frequently present. Because manual compression is impossible, unlike at a puncture site, it is preferable to use a stent graft that can be used for hemostasis, such as VIABAHN (W. L. Gore & Associates, Flagstaff, AZ, USA). However, this requires the insertion of a wire; if there is no wire, hemostasis can only be achieved by thoracotomy or laparotomy. Moreover, even with laparotomy, it may be impossible to secure the operative field due to massive bleeding because hemostasis has not been achieved. Therefore, it is essential to keep the wire and catheter inserted until just before the procedure is completed.

**Fig. 2:**
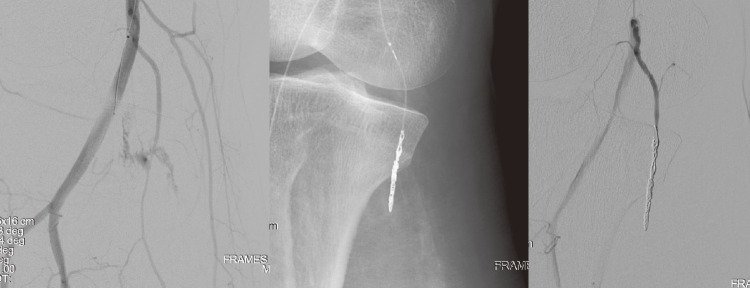
Post-procedural bleeding was identified in a branch of the popliteal artery, and coil embolization was performed for hemostasis.

In addition, vessel injury may occur due to the wire itself or high-pressure balloon expansion. Wire-related injury is usually relatively small, and there is some time margin. However, it can also cause compartment syndrome due to hematoma, and management is necessary. Hemostasis can be achieved by coil embolization or embolization with Spongel or N-butyl cyanoacrylate (NBCA), among others, for small vessels that are not problematic after hemostasis (**Fig. 2**). For bleeding in visceral arteries, hemostasis is achieved using a covered stent, such as VIABAHN, similar to stent grafts.

In a systematic review of complications of the femoral artery approach, bleeding that is difficult to control is reported to occur in 1%, whereas pseudoaneurysm is reported to be frequent at 2%–8%.^3)^ Other findings include arteriovenous fistula, dissection, peripheral embolism, and nerve injury. Procedural risks include sheath size of 8 Fr, high or low puncture, female sex, and severe calcification as contributing risk factors. In the early period, access complications with large-bore sheaths were reported to occur at a relatively high rate of 13%–22%,^4,5)^ but recently, they have been decreasing with low-profile devices.^6)^

## II. Vascular Dissection

Iatrogenic dissection is a complication encountered relatively often in EVT, similar to bleeding. Dissection of the access vessel is frequently encountered in thoracic stent grafting (thoracic endovascular aortic repair [TEVAR]) and abdominal stent grafting (endovascular aneurysm repair [EVAR]). The basic response is stent placement; however, it is often a retrograde dissection, and intimal fixation may be required when the entry is in the femoral artery. In addition, prosthetic graft replacement may be performed in complex dissection cases in which vessel repair is difficult. The frequency of access dissection in TEVAR or EVAR has been reported to be approximately 0.5%–5.0%.^3,4,6,7)^

**Fig. 3:**
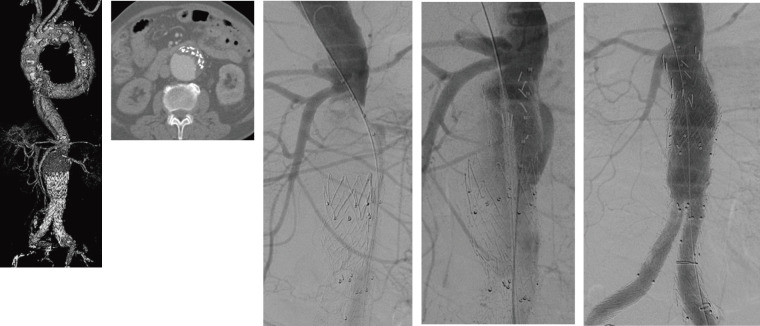
CT showed stent graft compression due to retrograde type B aortic dissection, and additional stenting was performed. CT: computed tomography

In TEVAR and EVAR, retrograde dissection caused by the stent graft can also be a problem. In particular, retrograde type A dissection occurs in 2.5% of patients after TEVAR, and its mortality rate is extremely high at 37%.^8)^ Although univariate, it is said to be more frequent with Zone 0 landing and with devices that have a proximal top stent. However, retrograde type B dissection is relatively rare, but case reports are sporadically seen^9,10)^; the detailed frequency is unknown, but it also tends to be somewhat more common with grafts that have a top stent (**Fig. 3**).

All of these are serious complications, and because they may occur intraoperatively and after some time postoperatively, postoperative follow-up is important.

The National Heart, Lung, and Blood Institute (NHLBI) classification, analogous to coronary artery dissection, is often used.^11,12)^ In peripheral vessels, dissections often do not become problematic and observation is sufficient; however, stent insertion is often performed in Grade D or higher in the NHLBI classification. Intravascular ultrasound is very useful for confirming dissection intraoperatively, but it is often used as an adjunct due to reimbursement issues.

## III. Covering Branches, etc.

Even if branches are accidentally covered with a normal bare-metal stent in the treatment of lower extremity arteriosclerosis obliterans, it is relatively uncommon for this to become problematic. However, covering branches with covered stents or grafts may cause ischemia, and caution is required. Relatively common situations include cervical branch coverage during TEVAR and abdominal branch coverage during EVAR.

In TEVAR, coverage of the left subclavian artery may cause symptoms such as arm fatigue if there is vertebral artery collateral flow, but it does not become a major problem in many cases. If necessary, the neck is immediately cut down, the common carotid artery is exposed, a sheath is inserted, and a wire is inserted retrogradely if the left common carotid artery is covered. If a wire can be inserted into the gap between devices, rescue is performed as a chimney technique by inserting a stent or stent graft into the carotid artery. If it is completely covered, rescue is performed by in situ fenestration, such as the retrograde in situ branched stent-grafting technique, or by placing a bypass. The carotid approach can also be performed by puncture; however, performing it by cutdown is preferable for preventing cerebral embolism.

The most problematic issue in EVAR is usually renal artery coverage. When the vessel condition is good and a device without a top stent is being used, wires are inserted into the right and left limbs and pulled down as a pull-through. However, forcibly pulling down may cause dissection or embolism. Therefore, especially when the neck condition is poor, it should not be performed, and insertion of a renal artery stent becomes the first choice. Renal artery stent insertion is usually easier after completing the stent graft completely because device migration is less and vessel alignment becomes better, making cannulation easier. The basic approach is to attempt cannulation from the ipsilateral CFA on the covered renal artery side using a renal double curve (RDC) catheter. If performing it from the ipsilateral CFA is difficult, approach from the brachial artery may allow easier passage.^13,14)^

Although it is not standard EVAR, the superior mesenteric artery may be covered in stent grafting for thoracoabdominal aortic aneurysm and the like, and because the ischemic time is extremely short, laparotomy should not be hesitated.

## IV. Embolism and Device-Related Complications

**Fig. 4:**
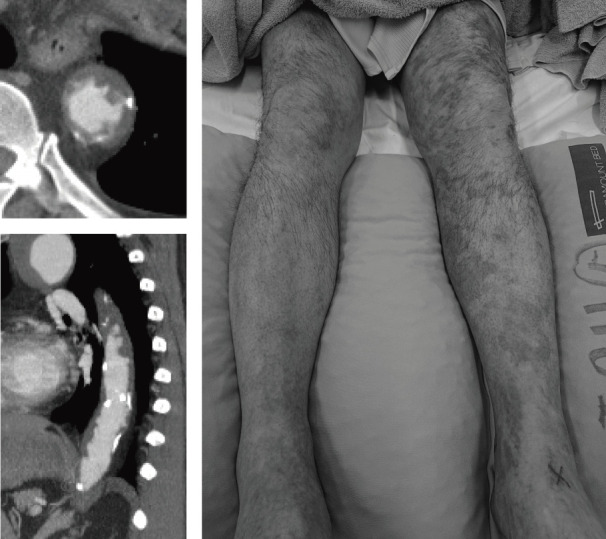
Distal embolization sometimes occurs in cases such as a shaggy aorta syndrome.

Intravascular plaque or thrombus may be scattered by wire or catheter manipulation in EVT. These are called embolisms. Fine emboli may not be removable and may only be observed, and blue toe syndrome and similar symptoms may occur. The risk of embolism is high in large vessels, in the so-called shaggy aorta in which thrombus is diffusely present on the aortic wall, and caution is required (**Fig. 4**). In TEVAR for shaggy aorta, embolism occurs in 7% of patients, and those who develop embolism have higher late mortality.^15)^

When emboli occur in the periphery and are recognized as clear defects on angiography, embolus removal with a Fogarty balloon is performed; if it is a thrombus, thrombolytic therapy or retrieval devices are used to attempt retrieval. If treatment is difficult, it is surgically removed by open surgery, or if localized, bail-out procedure may be possible by pressing it against the vessel wall with a balloon.

Complications related to the device itself, such as inadequate device deployment, may occur. Preventing device malfunction is difficult, and the best judgment must be made when it occurs. Small-caliber stents and coils may deviate or migrate and be inserted into unexpected locations. In particular, when a sheath or catheter passes through a previously placed stent, it may catch and cause device migration; if catching is a concern during insertion, devices should be carefully inserted under fluoroscopy.

**Fig. 5:**
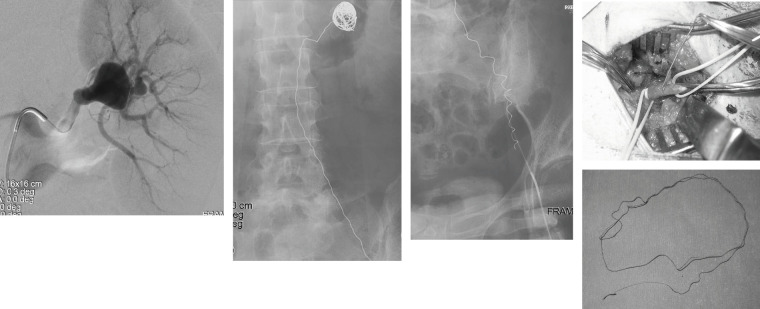
Coil embolization was scheduled to treat the left renal artery aneurysm. A coil was dropped out and flowed toward the femoral artery. Removal of the coil was performed with exposure of the femoral artery.

If a device cannot be expanded due to malfunction, a new balloon may be inserted over the wire to expand the device. If the device is in a non-deployed state, it can be stored in a large-bore sheath, and the insertion of a new device can be considered. Direct management may be required in situations such as partial deployment failure. If a part of the device (such as a distal tip) remains in the vessel, it can be guided out of the vessel and retrieved using a snare catheter. If it can be guided to some extent, the vessel can be directly exposed and removed under direct vision (**Fig. 5**).

## V. Infection

Infection includes puncture- or incision-site infection and late device infection. Incision-site infection is relatively rare and easy to manage with drainage, but device infection, such as of a stent graft, can be difficult to treat. Infection of a groin incision wound at the time of stent grafting is reported to be approximately 0.03%–3%.^16,17)^ Device infection is often fatal; however, if it is a simple infection, it may be manageable with antibiotic administration. In many cases, at least 6 weeks of intravenous antibiotic therapy is necessary, followed by long-term oral antimicrobial administration as maintenance therapy.

However, surgery is necessary when a gastrointestinal fistula (aorto-enteric fistula, AEF) occurs involving the gastrointestinal tract, such as the esophagus or duodenum. In this case, highly invasive surgery, including complete device removal, gastrointestinal resection, revascularization, and gastrointestinal reconstruction, is required. Regarding whether to choose anatomical revascularization (in situ) or extra-anatomical reconstruction after device removal for AEF after EVAR, the 2020 European Society for Vascular Surgery guideline recommends in situ reconstruction as the first choice.^18)^ An autologous vein graft, such as the deep femoral vein, or a rifampicin-soaked prosthetic graft is recommended. Rifampicin-soaked prosthetic grafts are not covered by insurance in Japan; therefore, they should be used in each institution under ethically considered circumstances.

In Japan, infection after EVAR was observed in 1.2% of patients during a mean follow-up period of approximately 44 months.^19)^ Removing all infected grafts is desirable, and it has been reported that the persistence of the infection source is also associated with late mortality.^18,20)^

## Conclusion

EVT is a remote operation, and direct management may be difficult when complications occur. However, in many cases, if a wire is in place, various methods can be used to manage the situation, and it is essential not to remove the wire until the procedure is completed. In addition, when it is judged that endovascular management is not possible, direct measures, such as laparotomy, should be performed without hesitation.
